# Cell death in cancer chemotherapy using taxanes

**DOI:** 10.3389/fphar.2023.1338633

**Published:** 2024-01-05

**Authors:** Ana P. Xu, Lucy B. Xu, Elizabeth R. Smith, Joshua S. Fleishman, Zhe-Sheng Chen, Xiang-Xi Xu

**Affiliations:** ^1^ Department of Biology, University of Miami, Coral Gables, FL, United States; ^2^ Sylvester Comprehensive Cancer Center, University of Miami Miller School of Medicine, Miami, FL, United States; ^3^ Department of Obstetrics, Gynecology and Reproductive Sciences, University of Miami Miller School of Medicine, Miami, FL, United States; ^4^ College of Pharmacy and Health Sciences, St. John’s University, Queens, NY, United States; ^5^ Department of Radiation Oncology, University of Miami Miller School of Medicine, Miami, FL, United States

**Keywords:** chemotherapy, taxanes, taxol, paclitaxel, microtubules, mitosis, proliferation, nuclear envelope

## Abstract

Cancer cells evolve to be refractory to the intrinsic programmed cell death mechanisms, which ensure cellular tissue homeostasis in physiological conditions. Chemotherapy using cytotoxic drugs seeks to eliminate cancer cells but spare non-cancerous host cells by exploring a likely subtle difference between malignant and benign cells. Presumably, chemotherapy agents achieve efficacy by triggering programmed cell death machineries in cancer cells. Currently, many major solid tumors are treated with chemotherapy composed of a combination of platinum agents and taxanes. Platinum agents, largely cis-platin, carboplatin, and oxaliplatin, are DNA damaging agents that covalently form DNA addicts, triggering DNA repair response pathways. Taxanes, including paclitaxel, docetaxel, and cabazitaxel, are microtubule stabilizing drugs which are often very effective in purging cancer cells in clinical settings. Generally, it is thought that the stabilization of microtubules by taxanes leads to mitotic arrest, mitotic catastrophe, and the triggering of apoptotic programmed cell death. However, the precise mechanism(s) of how mitotic arrest and catastrophe activate the caspase pathway has not been established. Here, we briefly review literature on the involvement of potential cell death mechanisms in cancer therapy. These include the classical caspase-mediated apoptotic programmed cell death, necroptosis mediated by MLKL, and pore forming mechanisms in immune cells, etc. In particular, we discuss a newly recognized mechanism of cell death in taxane-treatment of cancer cells that involves micronucleation and the irreversible rupture of the nuclear membrane. Since cancer cells are commonly retarded in responding to programmed cell death signaling, stabilized microtubule bundle-induced micronucleation and nuclear membrane rupture, rather than triggering apoptosis, may be a key mechanism accounting for the success of taxanes as anti-cancer agents.

## 1 Introduction

Several key cellular mechanisms of regulated cell death have been uncovered and studied. These are so-called programmed cell death: properly ordered processes triggered by well-regulated signaling pathways and discrete steps that often function in tissue homeostasis in physiological and immunological conditions. These cell death mechanisms are also considered to operate in the killing of cancer cells by chemotherapy. Taxanes, a key group of cancer drugs including paclitaxel, docetaxel, and cabazitaxel, are generally thought to bind and interfere with the dynamic of the cellular microtubules, leading to cell growth arrest, mitotic catastrophe, and ultimately caspase-3-mediated apoptotic programmed cell death. However, the precise mechanism of how taxanes trigger caspase activation is not established, and the cell death mechanism for taxanes in killing cancer cells is not clear. Here, we review the evidence and understanding for the cell death mechanism of taxanes in killing cancer cells and suggest that taxanes act by a newly reported mechanism, by facilitating the formation of multiple micronuclei (micronucleation) and the irreversible rupture of nuclear membranes.

## 2 Cell death mechanisms

In recent decades, several pathways of cellular events and mechanisms leading to cell death have been established (Wang, 2001; Liu and Lieberman, 2020). These pathways were revealed by both genetic and biochemical approaches to have elegant sequential steps in a regulated and ordered manner, referred to as programmed cell death (Hengartner and Horvitz, 1994; Metzstein et al., 1998; Sulston, 2003; Jiang and Wang, 2004; Linkermann and Green, 2014; Sun and Wang, 2014). The list of established mechanisms in executing cell death include apoptosis (Green and Reed, 1998; Wang, 2001; Chipuk and Green, 2008), necroptosis (Linkermann and Green, 2014; Sun and Wang, 2014), and perforin-mediated pore formation and membrane puncture (Cullen and Martin, 2008; McCormack et al., 2013; Podack and Munson, 2016). These cell death mechanisms participate in biological/developmental, physiological, and immunological processes.

In the apoptotic pathway, the mitochondria are punctured to release cytochrome c, which then activates the caspase cascade (Green and Reed, 1998; Wang, 2001). Cell death is the result of the caspase-mediated proteolytic destruction of proteins and cellular structures, as well as extensive cleaving of genomic DNA by activated endonucleases.

Necroptosis is facilitated by the activation and assembly of MLKL (mixed lineage kinase domain-like) protein to form channels on the nuclear and plasma membranes (Sun and Wang, 2014). Presumably, the puncturing of membranes and consequent leaking of nuclear and cellular components results in cell death.

Immune cells eliminate microbes and infected cells through granule/perforin-mediated membrane puncturing (Cullen and Martin, 2008; McCormack et al., 2013; Podack and Munson, 2016; Liu and Lieberman, 2020). In this process, immune cells recognize and release granules on the targeted cells. The activation of the released perforins (or other pore forming proteins) oligomerize and assemble into channels that insert onto the surface membranes, leading to calcium waves and the leaking out of cellular components to achieve cell death (Liu and Lieberman, 2020).

Additional newly discovered programmed cell death mechanisms include: ferroptosis in which accumulated iron and lipid oxidation trigger membrane rupture and cell death; and pyroptosis in which gasdermins form pores on the plasma membrane and cause cell death (Linkermann and Green, 2014; Green, 2019; Liu and Lieberman, 2020). These mechanisms are found to associate with certain neurodegenerative, cardiovascular, and kidney diseases. Furthermore, with a broader definition cell death mechanisms can be further diverse and complex (Park et al., 2023). For examples, autophagy can result in cell death if critical cellular components are digested, or autophagy can confer cells to become resistant to cellular stress and chemotherapy insult (Buzun et al., 2021; Zhang and Liu, 2021). Cell death caused by excessive production of poly (ADP-ribose) was coined as the cell death mechanism of “parthanatos” after Thanatos, the god of nonviolent deaths in Greek mythology (David et al., 2009; Wang et al., 2009; Huang et al., 2022).

We discuss the relevance of the key mechanisms in cancer chemotherapy below.

## 3 Cancer treatment/therapy

In cataloging common methods of cancer therapy, a (likely incomplete) list can be made, which includes radiation; drugs/agents that perturb signaling balance between apoptosis and survival; drugs/agents that interfere with cellular metabolism and cellular function; chemical or physical means that promote cell necrosis, such as cryo-, thermo- (cold, heat therapy), and histotripsy/ultra-sonics (ultrasound force); immunotherapy that targets and eliminates cancer cells; and finally chemotherapy that uses cytotoxic agents to cause both apoptosis and necrosis, though the mechanism(s) are still not clearly understood. These represent the main modes of biochemical and physical processes leading to cell death.

**FIGURE 1:**
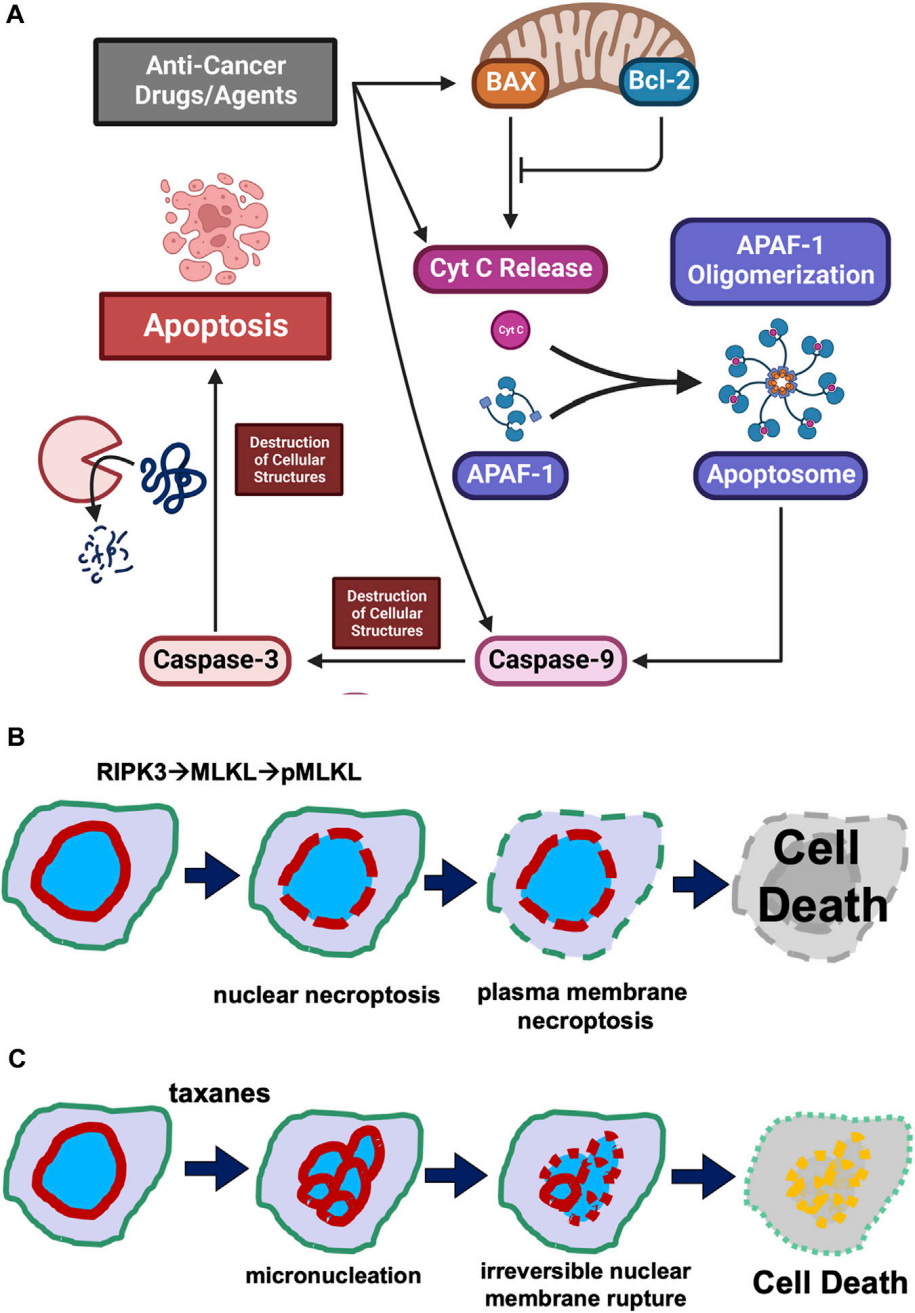
Cell death mechanisms. **(A)** Concept of the activation of programmed cell death pathway in cancer treatment. Illustration of the concept that anti-cancer drug/agents activate steps leading to activation of a step in the cellular regulatory pathway leading to programmed cell death is presented. It is generally considered that anti-cancer drugs/agents kill cancer cells by activating an intrinsic programmed cell death pathway, involving the leaking of mitochondrial cytochrome C, assembly of Apaf-1 into apoptosomes, activation of caspase 9 and subsequently caspase 3, leading to widespread proteolytic destruction of proteins and cellular structure, and ultimately cell death. **(B)** Puncture of plasma membrane and nuclear envelope in necroptosis. Necroptosis is a caspase-independent programmed cell death mechanism. Cellular signaling (such as cytokines) activates RIPK1 and then RIPK3 kinases, which phosphorylates MLKL. The phosphorylated MLKL polymerizes and assembles into a pore/channel that can insert onto the nuclear or plasma membrane, leading to membrane puncture and the leaking of nuclear or cellular components, ultimately causing cell death. **(C)** Taxane-induced irreversible membrane rupture. A more recent understanding is that paclitaxel (taxanes) kills cancer cell by inducing the formation of multiple micronuclei (micronucleation) and consequently causing the irreversible membrane rupture of the micronuclei. Taxanes induce the micronucleation and cell death by both mitotic and non-mitotic mechanisms.

It is a general notion that all cancer therapies, through either pharmaceutical agents or physical intervention, activate programmed cell death pathways to eliminate cancer cells (Figure 1A). Especially in targeted therapy, drugs/agents designed for a unique altered protein specifically in neoplastic but not in non-cancer cells, either directly or through multiple steps, lead to the activation of the apoptotic pathway, achieving the killing of cancer cells (Figure 1A). Presumably, anti-cancer drugs/agents activate a component in the upstream pathway, causing the leaking of mitochondrial cytochrome C into the cytoplasm to assemble apoptosomes. The result is the assembly of Apaf-1 into apoptosomes for the activation of caspase 9, and subsequently the activation of the effector caspase 3, leading to widespread proteolytic destruction of proteins and cellular structure, and ultimately cell death (Figure 1A).

However, cancer cells evolve to escape programmed cell death (Campbell and Leung, 2021; Hamilton et al., 2021; Neophytou et al., 2021; Hagenbourger et al., 2022; Zehnle et al., 2022), and this may be one of the several reasons that targeted or general cancer therapies have not yet gained overwhelming success.

Currently, cytotoxic chemotherapy is still the main frontline treatment for most cancer types (Holmes, 1996; Friedrich et al., 2004; Bookman, 2016; Lemstrova et al., 2016; Yang and Horwitz, 2017; Gallego-Jara et al., 2020; Yan et al., 2020). For taxanes, although no significant evidence has been documented, it is generally assumed that the stabilization of microtubules and the consequent aberrant mitotic event/mitotic catastrophe ultimately lead to the activation of the apoptotic cell death pathway (Inoue et al., 2001; Jordan, 2002; Bhalla, 2003; Jordan and Wilson, 2004; Zhao et al., 2022). Thus, the common notion is that taxanes, by binding and stabilizing microtubules, trigger caspase-mediated destruction of cellular components and ultimate cell death.

Lastly, immunotherapy to treat cancer has gained some successes and stimulated new excitement in recent years (Ribas and Wolchok, 2018; Esfahani et al., 2020; Perdyan et al., 2023). The new developments enable the engineering of immune cells targeting specific cancer antigens, and also provide strategies to active the immune system by blocking the ability of cancer cells to evade immune cells. The granule and pore forming machineries facilitate the killing of cancer cells by immune cells (Cullen and Martin, 2008; McCormack et al., 2013; Podack and Munson, 2016; Liu and Lieberman, 2020).

## 4 Caspase-mediated apoptosis

Classical programmed cell death is mediated by a signaling pathway to activate caspases, the proteinases that mediate the destruction of cells. The programmed cell death mechanism has been long observed in embryonic development and biological processes (Kerr et al., 1972). Many of the genes involved in programmed cell death (the *ced* genes) and also their sequential relationships were first identified using the model organism *Caenorhabditis elegans* (Metzstein et al., 1998; Sulston, 2003). The steps in the pathway were completed and understanding on a mechanistic level were further achieved by experiments using biochemical methods (Liu et al., 1996; Yang et al., 1997; Zou et al., 1997; Budihardjo et al., 1999; Jiang and Wang, 2004).

The central scheme is that cell-death signals trigger the formation of pores from Bax protein, which oligomerizes and inserts onto the mitochondrial membrane and facilitates the leaking of the electronic chain carrier cytochrome C (Green and Reed, 1998; Wang, 2001; Chipuk and Green, 2008) (Figure 1A). The Bcl2 protein acts as a decoy to bind and counter Bax, preventing puncturing of mitochondrial membrane by channels formed from the oligomerization of Bax (Ashkenazi and Dixit, 1999; Chipuk and Green, 2008; Bogner et al., 2010). Cytochrome C then seeds the formation of apoptosomes to facilitate activation of caspase 9 and subsequent caspase cascades (Budihardjo et al., 1999; Slee et al., 2001; Jiang and Wang, 2004). The activation of caspase 3, the so-called “executional caspase” with a wide substrate spectrum, leads to proteolytic destruction of the cells, which is the process of apoptosis (Slee et al., 2001; Jiang and Wang, 2004).

In addition to the mitochondrial-dependent pathway described above (Wang, 2001; Jiang and Wang, 2004), caspase 8 can be also activated following recruitment by transmembrane cytokine receptors to the cell surface, and subsequent activation of the caspase amplifying cascade leads to apoptosis in a non-mitochondrial-dependent mechanism (MacFarlane, 2003; Wang and El-Deiry, 2003).

For taxane-mediated chemotherapy, it is generally accepted that microtubule stabilization and subsequent mitotic catastrophe and micronucleation somehow cause cell death through apoptosis (McConley and Davis, 2002; Bhalla, 2003), though little evidences and details have been obtained to support the hypothesis or general understanding. Nevertheless, an enormous number of studies imply that cancer chemotherapeutics (including taxanes) induce apoptosis in tumor cells *in vitro*, and it has therefore been widely assumed that apoptosis is the mode of cell death in patient tumors exposed to these agents. However, there are also some outlier studies suggesting that taxanes do not kill cancer cells by the classical apoptotic pathway (Blagosklonny and Fojo, 1999; McConley and Davis, 2002). In a careful and critical review of literature, the involvement of caspase-dependent programmed cell death mechanism (apoptosis) in taxane chemotherapy is not as certain as it seems, as we will offer some thought and discussion below.

## 5 Necroptosis

A more recently established regulated (programmed) cell death mechanism is “necroptosis” (Linkermann and Green, 2014; Sun and Wang, 2014; Green, 2019), as opposed to the unregulated necrosis process. The necroptosis programmed cell death pathway is independent of activating caspases, instead involving MLKL (mixed lineage kinase domain-like protein) (Sun et al., 2012; Wang et al., 2014).

Upon stimulation, cytokine and death receptors activate the necroptosis pathway through a kinase cascade: facilitated by RIPK1 (Receptor-interacting serine/threonine-protein kinase 1) and RIPK3, leading to phosphorylation of MLKL (Sun et al., 2012; Wang et al., 2014). The phosphorylated/activated MLKL protein binds to the plasma membrane and oligomerizes to form pores or channels that perforate cell surface membranes, which causes cell death through swelling and hypotonic lysis (Linkermann and Green, 2014; Green, 2019; Liu and Lieberman, 2020) (Figure 1B). Cellular study also suggests that ESCRT-III is able to rescue necroptotic cell death by facilitating the shedding of MLKL-perforated areas of plasma membranes (Gong et al., 2017).

In addition to targeting the plasma membrane, necroptosis can also facilitate the perforation and rupture of the nuclear membrane using the RIPKs and MLKL components (Zhang et al., 2022) (Figure 1B).

In seeking the physiological roles for the necroptosis mechanism, the RIPK1-RIPK3-MLKL-dependent necrosis was found to promote aging of the male reproductive system (Li et al., 2017; Li et al., 2020), and is also involved in corpus luteum regression in mouse models (Li et al., 2021).

Several additional variations of the regulated cell death pathways have also been proposed, such as pyroptosis and ferroptosis (Linkermann and Green, 2014; Green, 2019; Liu and Lieberman, 2020). Similar mechanisms of perturbation of membrane integrity also operate in pyroptosis, in which pore forming proteins, gasdermins, are proteolytically activated, forming large pores on plasma membrane and causing cell death by leaking cellular content (Liu and Lieberman, 2020). Unlike apoptosis, necroptosis and these additional programmed or regulated cell death pathways generally result in the expulsion of cellular content and cause inflammatory reactions (Liu and Lieberman, 2020). These pathways are often engaged by immune systems. Currently, there is no strong evidence found to suggest necroptosis plays significant roles in taxane-mediated cancer therapy.

## 6 Granule- and perforin-mediated cell death

Immune cells target and kill microbes and infected or mutated host cells by granzyme- and perforin-mediated mechanisms (Cullen and Martin, 2008; McCormack et al., 2013; Podack and Munson, 2016; Liu and Lieberman, 2020).

Three pore-forming proteins of the mammalian immune system were discovered to operate in a redundant and also complementary manner to defend against invading or mutant cells (McCormack et al., 2013; Podack and Munson, 2016). Complement factor C9 functions in targeting and eliminating extracellular bacteria; Perforin-1 is tasked with dealing with intracellular viruses and cancer cells; and Perforin-2 can eradicate both intra- and extracellular bacteria (Cullen and Martin, 2008; McCormack et al., 2013; Podack and Munson, 2016; Liu and Lieberman, 2020).

Like necroptosis, perforin and granule-mediated cell death involves pore formation in the target cell membrane and the releasing of cell contents (granule and granzyme) to achieve cell killing. These mechanisms are employed in the immune system, including the nature killer cell and cytotoxic T lymphocyte (Liu and Lieberman, 2020).

Certainly, the granule and perforin-mediated mechanisms play essential roles in cancer immune therapies, though no involvement in chemotherapy has been clearly suggested.

## 7 Taxanes cause micronucleation and nuclear membrane rupture in both mitotic and non-mitotic cells

Despite the fact that taxanes are a key group of successful drugs used in frontline therapy as well as second line treatment of recurrent cancer for several major solid tumors, especially metastatic cancer (Yang and Horwitz, 2017; Yan et al., 2020), their cancer killing mechanism is still not clear (Blagosklonny and Fojo, 1999; McConley and Davis, 2002). Although the generally accepted notion is that taxanes inhibit mitosis or cause mitotic catastrophe by binding to microtubules and interfering their mitotic function (Yang and Horwitz, 2017), subsequently inducing apoptosis, the molecular pathway(s) to activate caspases has not been established (Blagosklonny and Fojo, 1999; McCormack et al., 2013).

A more recent understanding is that taxanes (paclitaxel) kill cancer cells by inducing the formation of multiple micronuclei (micronucleation) and consequently causing the nuclear membrane to irreversibly rupture (Smith and Xu, 2021) (Figure 1C). Taxane-induced micronucleation is facilitated by both mitotic (Weaver, 2014; Zasadil et al., 2014) and non-mitotic mechanisms (Smith et al., 2021; Smith and Xu, 2021; Smith et al., 2022). Conversion of a singular globe of nucleus into multiple micronuclei would greatly stretch the surface area, assuming the cell stays the same volume. For example, breaking up a sphere into 8 smaller spheres will double the surface area (the ration is expressed as: n/n^2/3^, n is the number of micronuclei assuming all are the same size).

The nuclear envelope has been observed to undergo rupture in cancer cells in culture (Vargas et al., 2012). The ruptured nuclear membrane often can be repaired (Gong et al., 2017). However, micronuclei, which are often defective, commonly undergo catastrophic corruption (Hatch et al., 2013). We postulate that the irreversible rupture of taxane-induced micronuclei is a key mechanism for paclitaxel cancer cell killing (Figure 1C) (Smith et al., 2021; Smith and Xu, 2021; Smith et al., 2022). In ovarian cancer, the expression of Lamin A/C protein appears to be a key determinant of paclitaxel responsiveness (Smith et al., 2021; Smith et al., 2022). Lamin A/C-low cancer cells readily undergo micronucleation with the presence of paclitaxel-induced microtubule bundles to facilitate nuclear envelope distortion and breaking (Smith and Xu, 2021). Previous studies have established that cellular Lamin A/C is lost or reduced in breast and ovarian cancer cells (Capo-chichi et al., 2011a; Capo-chi et al., 2011b; Capo-Chichi et al., 2016; Smith et al., 2018a; Smith et al., 2018b), and reduced Lamin A/C underlying nuclear envelope distortion and malleability (Smith et al., 2017), explaining the mechanism that Lamin A/C is a key determinant of paclitaxel sensitivity (Smith et al., 2021; Smith et al., 2022).

## 8 Summary and prospective

Cell death mechanisms in either regulated biological processes or cancer therapy seem to commonly involve targeting and compromising membrane integrity: mitochondrial channel formation from Bax oligomer in apoptosis (Bogner et al., 2010), MLKL pores formed on cell surfaces and nuclear membranes in necroptosis, or perforin pores inserted onto plasma membrane of targeted cells (Liu and Lieberman, 2020). Irreversible and severe damaging of cellular structures and organelles either by proteolytic destruction or pore-mediated puncture of membranes appear to be key manners in causing cell death (Liu and Lieberman, 2020).

**FIGURE 2:**
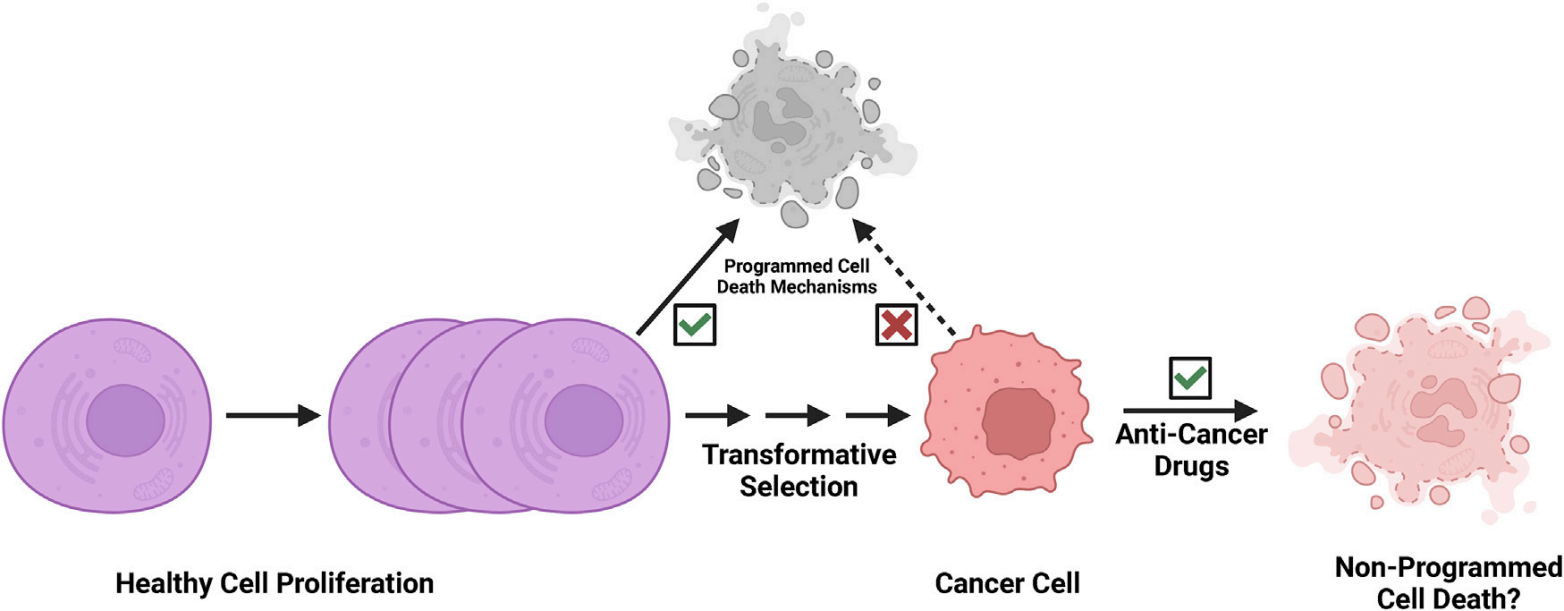
Cancer cells are refractory to programmed cell death. Programmed cell death pathways play key roles in tissue homeostasis and immune surveillance in development and physiological conditions. In the process of neoplastic transformation, cells multiply and are altered/mutated, but many altered cells that arise are then likely purged by programmed cell death mechanisms and immune surveillance. However, the malignant cells eventually arise following clonal evolution, and these persistent malignant cells become refractory to programmed cell death through selection. We speculate that effective cancer therapy likely exploits a non-programmed cell death mechanism.

Programmed cell death pathways play key roles in tissue homeostasis and immune surveillance in physiological conditions (Kerr et al., 1972). In the process of neoplastic transformation, cells multiplied and altered/mutated, but many altered cells that arose were likely purged by programmed cell death (Figure 2). However, the malignant cells eventually arise following clonal selection, and these persistent malignant cells become refractory to programmed cell death (Figure 2). Since cancer cells evolve to escape programmed cell death, and both apoptotic and necroptotic pathways are de-sensitized in the processes of transformation (Igney and Krammer, 2002; Mohammad et al., 2015; Campbell and Leung, 2021; Hamilton et al., 2021; Neophytou et al., 2021; Hagenbourger et al., 2022; Zehnle et al., 2022). Non-programmed cell death mechanisms may play important roles in strategies of cancer treatment (Figure 2). Thus, we speculate that the rupture of nuclear or plasma membranes by physical forces or biochemical events may be a more feasible mode of action in successful cancer therapy (Smith and Xu, 2021).

For taxanes that are effective even to Tp53 mutated and apoptotic refractory cancer cells, the rupture of the nuclear membrane may be an important mechanism. First, taxanes induce micronucleation through aberrant mitosis or a non-mitotic mechanism of nuclear membrane tearing. The micronuclei are compromised and undergo catastrophic membrane rupture, leading to cell death, which may be an unregulated and passive process (Smith and Xu, 2021). New understanding of such a mechanism may necessitate re-thinking strategies for cancer therapy and prompt development of suitable methods to enhance taxane efficacy and to overcome resistance.

## Data Availability

The original contributions presented in the study are included in the article/Supplementary material, further inquiries can be directed to the corresponding author.
